# Grid batch-dependent tuning of glow discharge parameters

**DOI:** 10.3389/fmolb.2022.910218

**Published:** 2022-08-18

**Authors:** Ramy Kazan, Gabrielle Bourgeois, Dominique Carisetti, Ileana Florea, Eric Larquet, Jean-Luc Maurice, Yves Mechulam, François Ozanam, Emmanuelle Schmitt, Pierre-Damien Coureux

**Affiliations:** ^1^ Laboratoire de Biologie Structurale de la Cellule, BIOC, Ecole Polytechnique, CNRS-UMR7654, IP Paris, Palaiseau, France; ^2^ Thales Research and Technology, Palaiseau, France; ^3^ Laboratoire de Physique des Interfaces et Couches Minces (LPICM), CNRS-UMR 7647, Ecole Polytechnique, IP Paris, Palaiseau, France; ^4^ Laboratoire de Physique de la Matière Condensée, Ecole Polytechnique, CNRS, IP Paris, Palaiseau, France

**Keywords:** cryo-EM, sample preparation, glow discharge, quantifoil, continuous carbon

## Abstract

Sample preparation on cryo-EM grids can give various results, from very thin ice and homogeneous particle distribution (ideal case) to unwanted behavior such as particles around the “holes” or complexes that do not entirely correspond to the one in solution (real life). We recently run into such a case and finally found out that variations in the 3D reconstructions were systematically correlated with the grid batches that were used. We report the use of several techniques to investigate the grids' characteristics, namely TEM, SEM, Auger spectroscopy and Infrared Interferometry. This allowed us to diagnose the origin of grid preparation problems and to adjust glow discharge parameters. The methods used for each approach are described and the results obtained on a common specific case are reported.

## Introduction

Electron microscopy of frozen hydrated samples (cryo-EM) is a powerful structural technique that allows the direct observation at a high resolution of functional macromolecular complexes in their near-physiological environment. To obtain high-resolution images, one needs first to vitrify a thin layer (ideally between 50 and 150 nm thickness) of a sample of interest. This task is not that straightforward, and many trials and errors are needed to obtain an optimal ice thickness (Cho et al., 2013). Sample buffer, sample concentration, temperature and humidity chamber, type of grids (Quantifoil, C-flat, and Holey), grid sample support (holey carbon, gold, nickel, and graphene), with or without an extra continuous carbon layer, glow discharge conditions, blotting time, and blotting force are parameters that are usually screened (Passmore and Russo, 2016) to improve grid preparation. Finally, the air–water interface diffusion also needs to be taken into account (Glaeser, 2018) as it also influences particle orientation and stability (Noble et al., 2018; Jahagirdar et al., 2020).

Recently, we observed that the same biological sample spotted onto similar grids coming from different batches led to dissimilar results. The biological sample is a ribosomal translation initiation complex (IC) from *Pyrococcus abyssi* containing two initiation factors aIF1A and aIF5B. The grid preparation was performed with this IC complex using Quantifoil R2/1 grids with 2 nm extra continuous thin carbon layer (TCL) as previously described (Coureux et al., 2016; Coureux et al., 2020). Ribosomal particles were clearly visible within all data collections. After data processing, the two factors of the IC complex were only visible in some cases. After addressing sample preparation/stability, grid preparation conditions, data collection parameters, or data processing strategy, we realized that the differences observed were linked to the batch of grids after our glow discharge conditions (−25 mA during 30 s). Those conditions were the same we used for previous batches of grids that led us to publish our cryo-EM results (Coureux et al., 2016; Coureux et al., 2020) and are consistent with the conditions widely used in the cryo-EM community. After further investigation, we hypothesized that, for some grid batches, the TCL was damaged during the glow discharge step (Lu et al., 2016) in our standard conditions (see Materials and Methods): the extra carbon layer seems of good integrity before glow discharge while it was completely sheared after. This observation was not the case for previous grid batches.

Glow discharge is an empirical method for rendering the carbon support film hydrophilic and, hence, facilitates the adsorption of biological material (Aebi and Pollard, 1987; Russo and Passmore, 2016). In brief, the grid is placed between two electrodes in a reduced atmosphere, usually of air. A high voltage is then applied to cause a glow discharge that generates an air plasma containing ions and radicals that, in turn, react with the carbon surface to reduce its hydrophobicity.

To further investigate the different behaviors of grids during glow discharge, several approaches have been used: overall grid quality was checked using Scanning Electron Microscopy (SEM). Carbon membrane thickness and composition were studied using electron energy loss spectroscopy (EELS) and Auger spectroscopy. Analysis of the surface chemical composition of the carbon membrane over time was performed on a study model using infrared spectroscopy. Local grid quality was also assessed using electron tomography (ET). Each technique, briefly presented, has its own limitations but can give simple and valuable information when one needs to better rationalize the quality of the thin extra continuous carbon membrane.

The first aim of this article is to report a case study where variations between grid batches were observed. The analysis of our cryo-EM experiments (data collection and processing) combined with the approaches mentioned earlier confirmed the importance of glow discharge adjustment depending on the grid batch to keep intact TCLs. The second aim of this article is to describe physical methods useful for cryo-EM grid characterization.

## Materials and methods

### Cryo-EM–Cryo-electron microscopy

#### Complex preparation

Translation initiation complexes from *Pyrococcus abyssi* (*Pab*) were prepared using a strategy similar to that previously described (Coureux et al., 2016; Coureux et al., 2020; Kazan et al., 2022)). In brief, the different partners of the complex were independently purified. The 30S *Pab* ribosomal subunits were extracted and purified from archaeal cells, and the initiation factors (aIF1A and aIF5B) and tRNA_i_^Met^ were over-expressed and purified from *E. coli*. The tRNA_i_^Met^ was then methionylated *in vitro* as previously described (Mechulam et al., 2007). mRNA was commercially synthesized. The full complex (called Initiation Complex 3 or IC3) was formed *in vitro* and purified. An excess of initiation factors (5x) was added, then the sample was incubated with the crosslinker BS^3^ (1 mM final concentration) for 10 min at 51°C just before spotting onto grids to favor complex formation (Querido et al., 2020). The final concentration of the full complex was 120 nM. After IC3 *in vitro* assembly, the sample was kept at 51°C shortly prior to grid preparation.

#### Grid preparation

In our study, we used three different batches of copper Quantifoil grids 300 mesh R2/1 grids with 2 nm TCL, named batches #1, #2, and #3. One batch of copper Quantifoil grids 300 mesh R2/1 with 3 nm TCL (called batch#4) was also used in this study. Each grid coming from batch#1, #2, or #3 was first glow-discharged (GD) with a Pelco Easiglow device set up at −25 mA for 30 s as routinely used (Coureux et al., 2020). The grid was then transferred within minutes into the LEICA EM-GP grid plunger. The chamber was set to 20°C and 90% humidity. Liquid ethane was maintained at −182°C and the preblot, blot, and postblot parameters for grid preparation were set to 10 s, 1.2 s with auto-sensor, and 0 s, respectively. 3.4 µL of the IC3 complex was applied onto the grid prior to the preblot. As a control for biophysical approaches, grids from batch#4 were also glow-discharged using the same preparation conditions. The summary of usage for each grid batch with the corresponding biophysical approach used is summarized in Table 1.

**TABLE 1:** Grid batch usage for each biophysical approach. Batches #1, #2, and #3 are copper Quantifoil R2/1 grids with a 2 nm extra continuous thin carbon layer. Batch #4 is copper Quantifoil R2/1 grids with 3 nm extra continuous thin carbon layer. Each batch was used (represented by an “x” sign) or not used (empty box) for some approaches mentioned in the first column. The infrared spectroscopy analysis was not performed on grid batches (explained by the n.d. (not determined) abbreviation) as a Si prism was used.

<--col count 2-->	
																	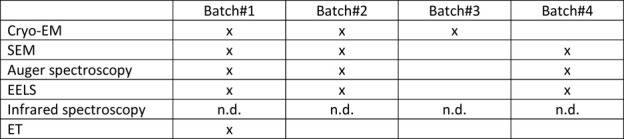	

#### Data collection and processing

Cryo-EM data were recorded either on the in-house TFS Titan Themis (X-FEG operating at 300 kV) microscope equipped with a Falcon 3 camera and a Gatan ELSA holder at the CIMEX facility at Ecole polytechnique or on the TFS Titan Krios (X-FEG operating at 300 kV) microscope equipped with an energy filter (Bioquantum) and a Gatan K3 camera at Pasteur Institute in Paris. Total exposure of 40 e^−^/Å^2^ was applied to the sample and distributed on 39 or 40 frames on the corresponding camera. The final pixel sizes were 1.12 Å/pixel and 0.86 Å/pixel for the Themis and Krios data collection, respectively. Data processing of Single Particle Analysis (SPA) data was carried out with Relion 3.1 (Scheres, 2012) from raw images to the 3D structure. A motion correction of the frames recorded was performed using Relion’s own implementation of Motioncor2 (Zheng et al., 2017). CTF estimation, resolution limit, and astigmatism were estimated using gCTF (Zhang, 2016). Several rounds of 2D classification, 3D refinement, 3D classification, and final 3D refinement of the best-resolved 3D classes were performed with the Relion suite. In each case, the 3D classes with density corresponding to the IC3 complex were selected for further processing.

#### Scanning electron microscopy

Scanning electron microscopy was performed with an S4800 Hitachi FESEM for the observation of the surface topography of the carbon foil from the Quantifoil grids with 2 or 3 nm extra continuous carbon layer. This microscope was a cold field emission gun high-resolution scanning electron microscope (Accelerating Voltage from 0.5 to 30 kV), which is mainly based upon the detection of secondary electrons emerging from the surface under the impact of a very fine beam of primary electrons that scans the surface observed. The first interest of this type of microscope is its large range of magnification from X20 to X800K. The combined use of the super ExB filter with the first upper detector allows filtering and collecting secondary electrons and backscattering signal energies of interest, thereby suppressing charging artifacts and showing topographical details. In our case, all observations and images were made at 1 kV using the upper detector that guarantees a spatial resolution of 2 nm in conventional conditions.

#### Electron energy loss spectroscopy

The membrane thickness of Quantifoil grids with 2 or 3 nm extra continuous carbon layer was measured using EELS. No glow discharge was performed to prevent shearing the extra carbon layer. The microscope used was a transmission electron microscope (Jeol 2010F) equipped with a Gatan TRIDIEM imaging filter allowing for electron energy loss spectroscopy (EELS) and the recording of filtered images. The accelerating voltage was set to 200 kV. The thickness measurement was performed using the so-called log-ratio method, by comparing the intensity in an image recorded with all the transmitted electrons (the integral of the EEL spectrum, including elastic and inelastic electrons) with one recorded with only elastic electrons (the integral of the zero-loss peak). See Supplementary Figure S1 for details.

Let us call *I*_0_ the latter and *I*_tot_ the former; the ratio of the two depends exponentially on the sample thickness *t* through the following expressions:

$$I_{\mathrm{tot}}/I_{0}=\exp\left(t/\lambda \right),$$ (1)

where *λ* is the inelastic mean free path in amorphous carbon. Thus, the sample thickness is simply

$$t=\lambda \;\mathrm{Log}\left(I_{\mathrm{tot}}/I_{0}\right).$$ (2)

The inelastic mean free path *λ* depends on the material and measurement parameters (Malis et al., 1988; Iakoubovskii et al., 2008a; Egerton, 2009). The latter is the kinetic energy of the incident electrons and the convergence angle of the beam and the acceptance angle of the detector. However here, we recorded *t*/*λ* maps in imaging mode at an energy of 200 keV, using the software installed in Gatan Digital Micrograph. In this mode, the acceptance angle is so large that the whole scattered intensity can be considered collected (Iakoubovskii et al., 2008a; Iakoubovskii et al., 2008b). In such conditions, the inelastic mean free path in carbon is well documented in the literature (Malis et al., 1988; Iakoubovskii et al., 2008a; Iakoubovskii et al., 2008b; Egerton, 2009). It is for a thickness range close to the mean free path (70–150 nm), where the main energy loss mechanism is that of bulk plasmons, and the second one is that of the 1s core level ionization. Here, surface plasmons, which can be neglected in the reference configuration, dominate the other contributions.

Supplementary Figure S1 shows the comparison of a reference spectrum, recorded in the nearby thicker area (made of the Holey carbon membrane and the 2 or 3 nm extra continuous carbon layer) to a spectrum recorded on the TCL (made of the 2 or 3 nm extra continuous carbon layer only). Note the bulk plasmon peak at 24 eV (Berger et al., 1988) in the reference spectrum, is not visible in the TCL spectrum. The plasmon maximum appears to shift towards low energy in the latter case by more than 5 eV; which denotes the influence of surface plasmons, the energy of which has been positioned between 14 and 20 eV by X-ray photoelectron energy loss spectroscopy (Godet et al., 2009).

Quite counterintuitively, the total energy loss is proportionally larger for the present very thin films, as the energy loss mechanisms at work in thicker samples are not suppressed, and must add up to surface plasmons to make the total loss. Thus the actual inelastic mean free path must be significantly shorter in those very thin films. As developing a model taking the surface plasmons into account is out of the scope of the present work, we used the mean free path measured in diamond in the same conditions as the present ones by Iakoubovskii *et al.* (λ = 112 nm) (Iakoubovskii et al., 2008b), to which we applied a correction to take into account the change of density between diamond and amorphous carbon. For the dependence of *λ* upon density *ρ*, we used the expression in *ρ* ^*−*0.3^ given by the same authors (Iakoubovskii et al., 2008a), and for the density of amorphous carbon, we took that measured by Iwaki (2.15 ± 0.15 gcm^−3^) (Iwaki, 2002). With a density of diamond at 3.5 gcm^−3^, it gives *λ*_aC, 200kV_ = 130 nm. In this way, we got over-estimated values, which still can give useful indications.

#### Infrared spectroscopy

The sensitivity of infrared spectroscopy is too limited for the direct investigation of a carbon membrane deposited on a TEM grid. Therefore, a geometry of attenuated total reflection (ATR) has been used. For that purpose, a thin (1 nm) carbon film has been deposited on a ∼15 × 20 mm^2^ Si prism. The film mimics the TEM membranes with a much larger surface area. The prism was cut from a 0.5 mm thick Si wafer (FZ-purified, ∼10 Ω cm Si crystal). The two long parallel edges were polished at 45° using first abrasive papers of successive grades (from 240 to 1,200), then diamond pastes of successive grades down to 1 µm. The obtained prism finally provided 14 to 15 useful internal reflections for the infrared beam on the large face of the prism. Before use, final cleaning of the sample was performed by immersion for 15 min in a hot “piranha” solution (1:3 volumes of H_2_O_2_:H_2_SO_4_), leaving a clean thin oxide at the surface of the prism.

A thin carbon film was evaporated on the large face of the prism using a Cressington 208C. Two pulses of 6 s at 4.6 A gave a carbon thickness that corresponds to the perforated carbon deposited on copper grids.

The infrared spectra were recorded using a Bomem MB100 Fourier-transform infrared spectrometer coupled to an external homemade ATR compartment. After mounting the sample, the compartment was purged with N_2_ for 1 h before recording the data, in order to minimize the contribution of atmosphere water and carbon dioxide in the spectra. Each spectrum corresponded to the average of 400 acquisitions at a resolution of 4 cm^−1^. The infrared beam was polarized by passing through a wire-grid polarizer, and spectra in p and s polarizations were successively recorded without breaking the purge. Spectra are displayed in terms of absorbance scaled to one reflection. Absorbance was computed from the natural logarithm of the ratio of the considered spectrum intensity to that of a reference spectrum. Unless otherwise indicated, the reference spectrum is that of the sample prior to the application of the glow-discharge treatment.

Glow discharge of the Si prism was made with a Pelco EasiGlow device, using -25 mA for 30 s at 0.39 mbar. New infrared spectra were recorded at 1 and 4 h after glow-discharging to assess the evolution of the charges on the Si prism surface.

#### Electron tomography

For the tomography studies, unidirectional acquisition of the tilt series was performed in the classical Bright-Field (BF) mode of a Titan-Themis 300 microscope, using the serialEM (Mastronarde, 2005) acquisition software and a 4096 × 4096 pixels cooled CMOS Ceta camera. The data collection of the ET series was performed on Quantifoil grids with 2 nm TCL (batch#1). The same batch grids with or without glow discharge were analyzed twice for each condition. The tomography software allows an automatic variation of the tilt angle step by step, a correction of the focus of the image, and the preservation of the object under study within the field of view. The tilt angle was varied in a range from −60° to +50°, with an image recorded every 1° giving a total of 111 images with a total acquisition time of about 15 min. The data treatment of the tilt series for the preliminary image processing procedure was performed using the IMOD software (Mastronarde, 1997). The volume reconstruction was obtained using 50 iterations of the algebraic reconstruction technique algorithm (ART) (Gordon et al., 1970) implemented in the 2.8 version of the TomoJ software (MessaoudiI et al., 2007).

## Results

Cryo-EM–3D reconstructions of different datasets were performed to assess the *Pab* IC3 complex integrity. Depending on the dataset, the 3D reconstructions showed either the studied full complex IC3 or the IC3 complex lacking the initiation factors aIF1A and aIF5B. The resolution limits obtained in the different datasets varied around 3 Å (see FSCs in Supplementary Figure S2) and were sufficient to conclude the presence or absence of translation initiation factors, even at low thresholds.

**FIGURE 1:**
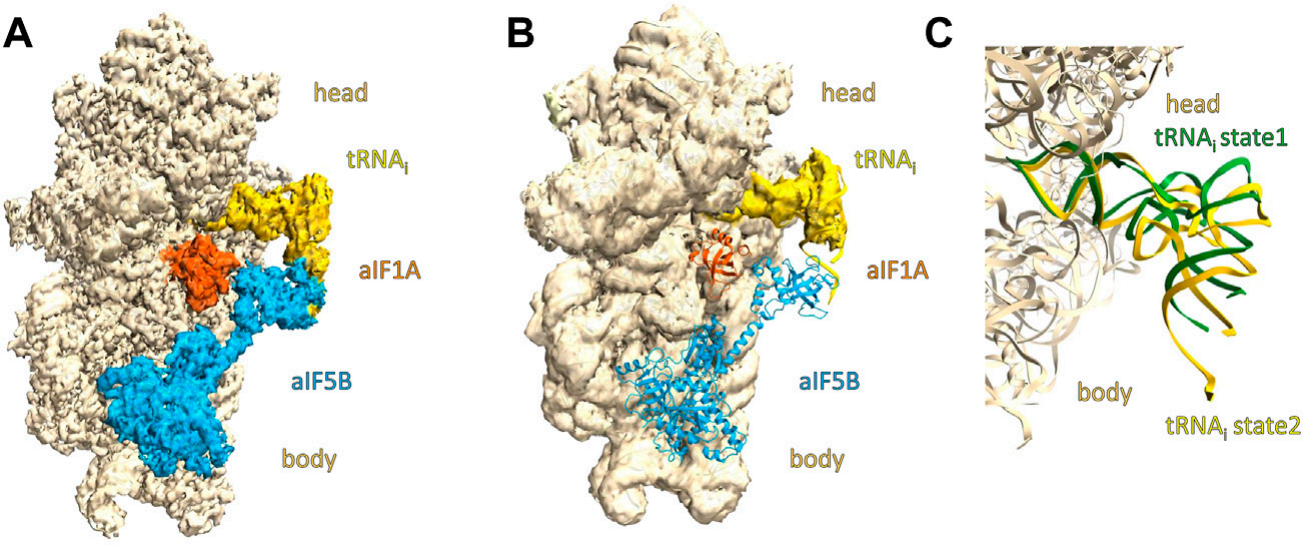
Overall view of cryo-EM maps of IC3 complexes prepared on grids coming from batch#3 and batch#1. The small ribosomal subunit 30S (made of the head and body parts) is colored in wheat, the tRNA is colored in yellow, aIF1A in orange, and aIF5B in cyan. 1A/3D density and corresponding model in the density of the IC3 complex containing the translation initiation factors aIF1A and aIF5B. 1B/3D density of the IC3 complex and corresponding model lacking density for translation initiation factors aIF1A and aIF5B. 1C/Close-up showing the tRNA_i_ conformations observed in structures 1A and 1B. The view was obtained after superimposition of the tRNAi conformation of the two structures. The model for aIF1A and aIF5B have been omitted for better clarity. The tRNA_i_ corresponding to the structure obtained in the IC3 complex lacking the translation initiation factors aIF1A and aIF5B is colored in green. The tRNA_i_ corresponding to the structure obtained in the full IC3 complex is colored in yellow. The tRNA in green is in an upper position compared to the tRNA colored in yellow.

Processed data from batch#3 (2 grids in total) gave 3D reconstructions where the potential map was clearly visible for the two initiation factors (Figure 1A). Processed data from batches #1 and #2 (5 grids in total), using the same sample and preparing the grids in the same experimental conditions, gave systematically stripped complexes without any density in the 3D reconstructions for the initiation factors (Figure 1B). Any further 3D classification did not help to identify a subset of particles showing the IC3 full complex. Comparing the experimental details we used for grid preparation, we noted a systematic correlation between the grid batch and the 3D reconstruction: the sample preparation and the grid preparation were as much as possible the same (see Materials and Methods).

When comparing the potential maps obtained, the major difference is obviously the presence or absence of aIF1A and aIF5B as well as tRNA_i_ conformations (Figure 1C). The position of the tRNA_i_ in the P site of the small ribosomal subunit in the stripped complex is rather in an upper position compared to the full IC3 complex.

To further investigate these results, different biophysical approaches were used to better characterize the different grid batches. Batch#4 was used as a control as grids from this batch did not show any morphological difference before and after glow discharge, and because we ran out of grids for batch#3.

**FIGURE 2:**
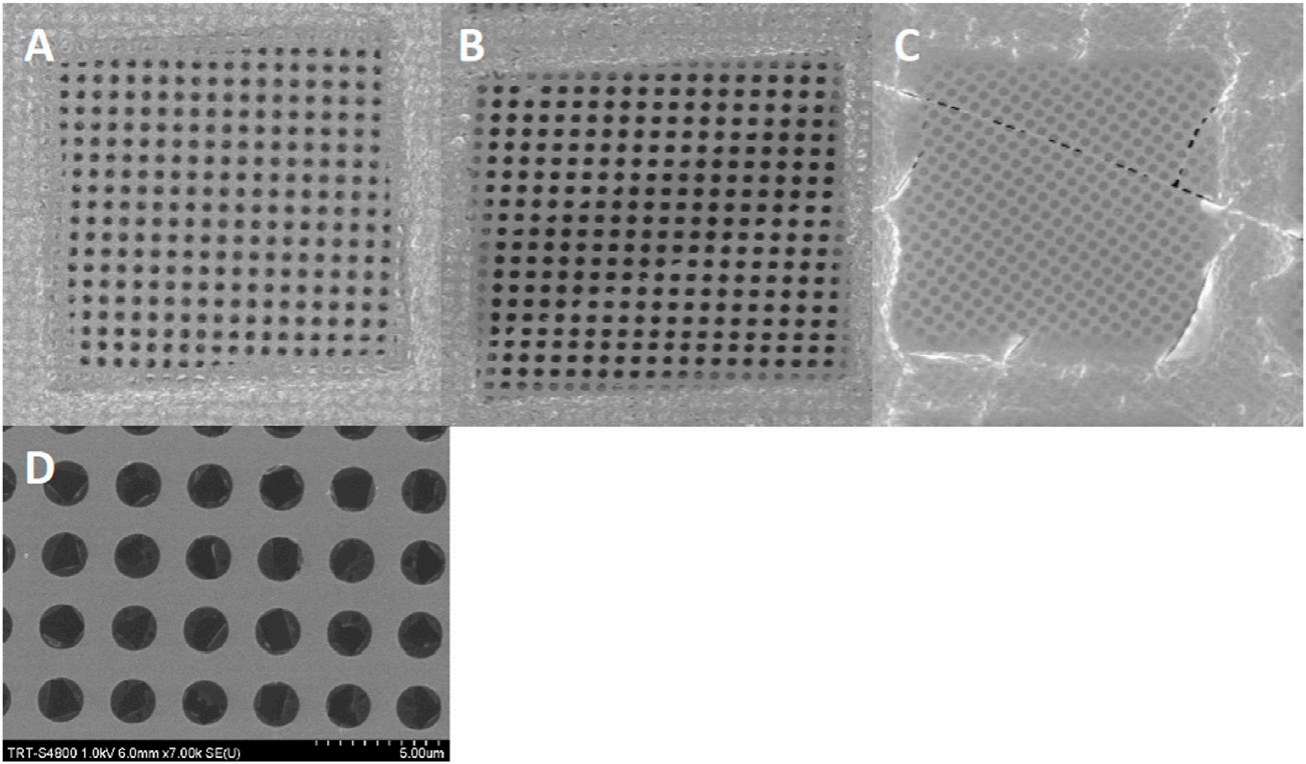
SEM images of a Quantifoil 300 mesh carbon grid with a 2 nm continuous carbon layer. A quick look at the grid with simple binoculars or SEM at low magnification (x700) doesn’t show clear defects in the grid (Figure 2A). A more careful analysis of the grid displays patches where the extra carbon layer is sometimes missing (Figure 2B) or contains irregular holes (Figure 2C). At higher magnification (x7000), the extra carbon layer is disrupted in each hole. (Figure 2D).

SEM–Bare grids from batches #1, #2, and #4 were used for SEM analysis. Each grid, with or without glow discharge, was carefully checked using conventional SEM imaging. For the nonglow discharged grids, at low magnification (×700), looking at whole grid squares, the carbon membranes seemed to be of good quality (Figure 2A). Some grid squares contained sheared regions where the extra thin continuous carbon layer was absent (Figures 2B, C). Going to higher magnification (7000x), for the glow discharged grids from batches #1 and #2, it became obvious that the TCL was broken, at least at the level of the holes of the perforated carbon layer (Figure 2D). This was not the case for the grids coming from batch#4, with or without glow discharge. Furthermore, Auger electron spectroscopy was also performed on nonglow discharged grids from batches #1, #2, and #4 to prevent the carbon layer on top of the perforated carbon to break. This analysis did not show a significant difference in chemical composition (Supplementary Figures S3, S4).

**FIGURE 3:**
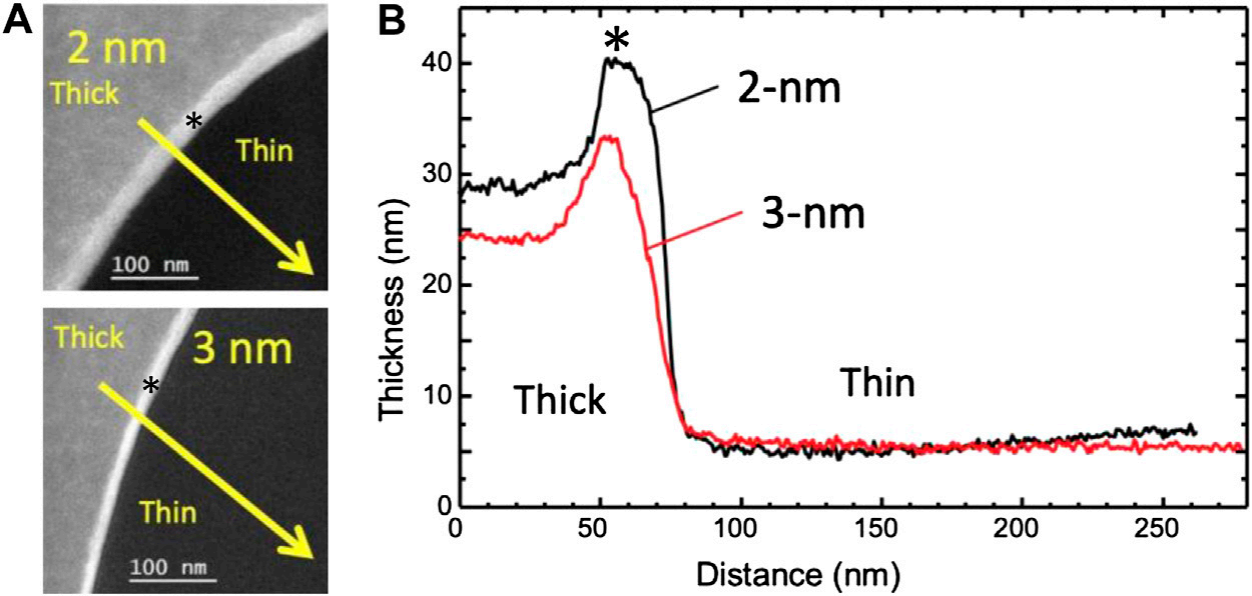
Thickness maps of Quantifoil 300 mesh carbon grid with 2 or 3 nm continuous carbon layer **(A)** Thickness maps were recorded as explained in the text, in a region such as that squared in Supplementary Figure S1A, on a 2 nm membrane (A1) and a 3 nm membrane (A2). **(B)** Thickness profiles were recorded along the yellow arrows in **(A)**, where the inelastic mean free path *λ* has been set to 130 nm: 2 nm nominal thickness in black, and 3 nm in red. The thickness maximum at both thick part edges is partly an artifact due to the presence of a Fresnel fringe (represented by the sign *).

EELS–Bare grids from batches #1, #2, and #4 were also used for EELS analysis. We measured the thickness of two TCLs with respective nominal thicknesses of 2 and 3 nm. The measurements were performed in five different areas in either case, taking 130-nm as the value of the inelastic mean free path λ. The raw data is available in Supplementary Table S1. The geometry of the measurements and the local thickness variations are shown in Figure 3.

If we assume that the differences with the nominal values are entirely due to the error on the mean free path, the actual inelastic mean free path would be shorter than the one used by respectively 58 and 44% for the 2 and 3 nm cases, which is consistent indeed with surface plasmons playing a lower role in the thicker samples.

Finally, given the specificity of the plasmon losses in thin membranes, we thus estimate that the measured values may be compatible with the nominal values of 2 and 3 nm. However, even if its average thickness is close to the nominal value, the 2 nm membrane presents more thickness variations than the 3 nm membrane.

**FIGURE 4:**
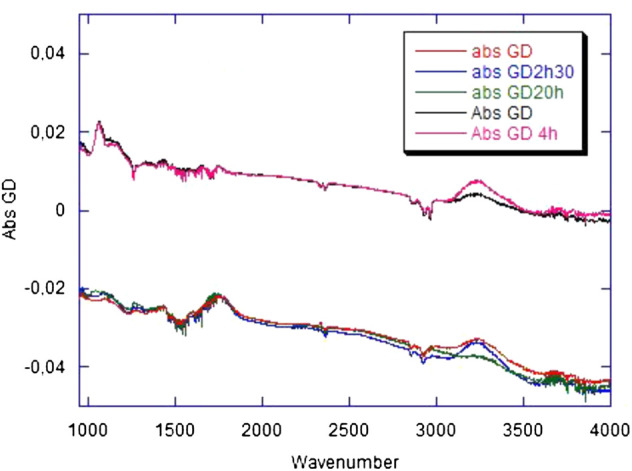
Infrared spectra of bare and carbon-covered Si prism. The red, blue, and green spectra (group in the lower part of the frame) correspond to the Si prism covered with a continuous carbon layer to simulate a classic Quantifoil grid; they have been recorded 1 h, 2 h 30 min, and 20 h after glow discharge, respectively. The black and pink spectra (upper part of the frame) correspond to the bare Si prism. Spectra have been recorded 1 and 4 h after glow discharge. All spectra have been recorded for an s-polarized infrared beam. In each case, the reference spectrum has been recorded prior to the GD treatment. The spectra are plotted as absorbances computed using a natural logarithm and correspond to 14 useful reflections in internal-reflection geometry.

Infrared spectroscopy–Figure 4 shows the absorbance changes induced by the GD treatment on the carbon film, about 1 hour after the treatment and at later times. As a reference, the absorbance changes observed for a bare Si prism submitted to the same treatment are plotted on the same figure. The plots represent the difference spectra taking the nonglow discharge material as the reference. Absorbance spectra exhibit vibrational peaks, positive when they result from the generation of chemical species by the treatment, and negative when they result from the removal of chemical species by the treatment. They also exhibit a baseline that, in the case of a bare Si prism, is characteristic of free-carrier absorption. It results from the change of the surface charge which affects the band bending in silicon close to the surface (Rao et al., 1986). The positive background of the spectra refers to a decrease in the space-charge region in silicon, corresponding to a positive charging of the surface. In the bare Si case, vibrational features evidence 1) the removal of adventitious carbon contamination (νCH_2_ and νCH_3_ modes around 2,900 cm^−1^ and δCH_3_ at 1,260 cm^−1^); a slight increase in the silicon oxide thickness (νSiOSi modes at 1,075 and tail in the 1,100–1,200 cm^−1^ range); 3) a hydroxylation of the silicon oxide surface (νOH mode around 3,250 cm^−1^). The first two features are expected phenomena upon treatment in an oxidative plasma. The third one reveals that the oxide surface tends to be covered by an increased amount of SiOH groups, plausibly resulting from the breaking of Si-O bonds upon plasma exposure, with the capture of protons coming either from water physisorbed at the surface or from atmospheric water in the plasma.

In the case of the carbon film, the baseline is of the opposite sign and exhibits a spectral shape distinct from classical free-carrier absorption (Drude-type absorption). These features suggest that the baseline can be tentatively assigned to a change in the charge of the surface, like for bare silicon, but which mostly affects the carbon film and not the underlying silicon oxide. This assignment is consistent with the absence of modification in the silicon oxide thickness (absence of the characteristic bands in the 1,050–1,200 cm^−1^ wavenumber range). However, the amplitude of the baseline appears to vary from experiment to experiment and may become null. Therefore, no reproducible specific charging of the carbon film can be detected. Two specific vibrational features appear and remain stable after the GD treatment: a broad asymmetric peak in the 1750 cm^−1^ range and a weaker one at ∼1,450 cm^−1^. The former evidences the combined formation of heavily oxidized carbon species (ketones, carboxylic acids, esters) and C=C bonds. Local aromatization could account for the presence of the 1,450 cm^−1^ peak. These peaks witness the irreversible changes induced by the GD treatment: breaking of C-C bonds and strong oxidation of the carbon film surface. A set of three other peaks are also induced by the treatment (a broad band centered at ∼3,250 cm^−1^, a relatively narrow peak at ∼1,290 cm^−1^, and a broader one around ∼1,100 cm^−1^), but they are seen to evolve on a time scale of several hours after the treatment. They all increase in the first hours after the treatment; at subsequent times, the 3,250 cm^−1^ band decreases, and the other ones keep growing. The 3,250 cm^−1^ band is assigned to the νOH mode of carboxylic-acid and alcohol type species. The bands at 1,290 and 1,100 cm^−1^ could mostly come from ester-type species.

An emergent picture consistent with the experimental observation is the following. The GD treatment induces the breaking of C-C bonds in the carbon film. The oxidative character of the plasma induces the irreversible formation of oxidized species and C=C bonds. However, some of the plasma-induced species remain unrelaxed and slowly evolve, inducing an increase in the population of surface species like alcohols and carboxylic acids. In a later stage, the layer relaxes, resulting in the formation of ester-type species resulting from the reaction of carboxylic acid sites with neighboring alcohol ones. Obviously, this picture remains schematic, and further specific mechanisms likely contribute to the phenomena. But whatever the detailed picture is, the slow evolution of the vibrational peaks after the end of the GD treatment points out that reactive species are induced by the exposure to the plasma, and that these species slowly relax on a several-hour time scale, affecting the reactivity of the carbon film surface. As a final remark, one may notice that a similar phenomenon (slow post-treatment evolution of the νOH band) takes place on the bare Si prism. Of course, in that case, carbon-specific species are not generated and detected, and the picture is likely simpler than for the carbon film. Here, the oxide surface deactivation corresponding to the slow evolution is ascribed to the formation of SiOH bonds arising from the H capture by non-bridging SiO species, a picture consistent with the decrease of a small peak at the low-energy side of the spectrum assigned to non-bridging SiO species.

### Electron tomography

**FIGURE 5:**
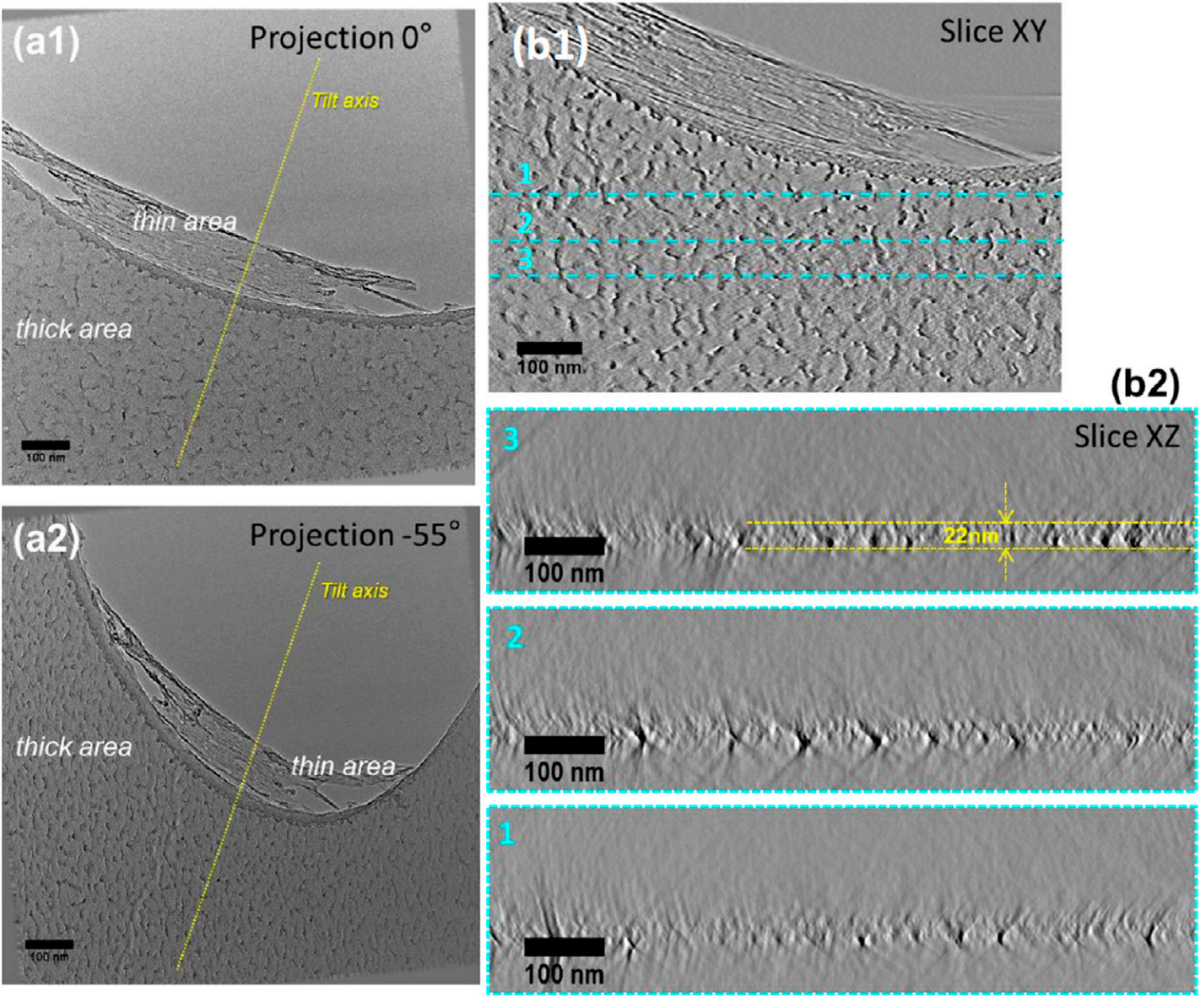
Electron tomography analyses of an area of the Quantifoil membrane containing a thick and thin area. **(A1)** and **(A2)** Projections acquired at two different tilt angles (−55° and +0°) from the tilt series used to calculate the reconstruction. **(B1)** and **(B2)**. Transversal and longitudinal slices were acquired at different depths and orientations through the reconstructed volume, illustrating the roughness of the Quantifoil membrane.

In order to reveal the structure of the Quantifoil membrane with respect to the initial structure and to confirm the assumptions made from the 2D-SEM and EELS observations as illustrated in Figures 2, 3, electron tomography (ET) analyses were performed on several areas within the membrane having undergone the same treatment as those illustrated in Figures 2, 3. Figure 5a1a2 displays two representative 2D-TEM micrographs of the analyzed area extracted from the tilt series used to reconstruct the volume, on which one can easily identify two different contrast regions corresponding to the thick and thin areas, respectively, with a marked apparent roughness. As a control (Figure 5a3), the same procedure was performed on a nonglow discharged grid. The ET analysis was performed twice on grids coming from batch#1 with and without glow discharge (Supplementary Figure S5): the pattern found on the grids from batch#1 with (thin area sheared) or without glow discharge (intact thin area) was identical. Also, the power spectrum of thick or thin areas was calculated to confirm the presence or absence of a carbon layer (Supplementary Figure S6).

By analyzing the projection series recorded one can easily observe that the structure of the thick area exhibits a marked difference at high tilt angles with respect to that at low tilt angles. A more detailed analysis of the projections recorded at low tilt angles, on which the presence of the two contrasts is clearly visible, allows us to unambiguously conclude, that the membrane has some granular species on both sides with different sizes that can be estimated to be about 7–10 nm. Representative sections through the volume of the reconstructed area are presented in Figure 5B. A quick visualization of the volume slice by slice clearly points out a rough structure of the membrane. This important observation is even more obvious when the volume is visualized in the direction perpendicular to the membrane axis (slices XZ1, XZ2, XZ3). We can clearly observe that the membrane is not perfectly flat, as is suggested by the 2D projections. Also, very importantly, the thickness of the membrane can vary between 20 and 25 nm. Regarding the thin part inside the hole, the ET analysis revealed that this part of the membrane is completely altered after glow discharge and falls back on the hole walls.

This ET study highlights the irregular structure of the Quantifoil membrane as well as its external roughness. A similar study is presented in the Supporting Information part on a larger area evidencing the same features of the membrane. A closer analysis of the thin area from the reconstructed volume of the series presented in Supplementary Figure S6 allowed us to estimate its thickness which is about 3 nm (for grids coming from batch#1).

## Discussion

The analysis of the different 3D reconstructions obtained from the same biological sample strongly suggested that grid batches can be an issue when performing cryo-EM experiments. The sensitivity of the 2 nm carbon layer to glow discharge was shown to vary between grid batches. Grids coming from batch#3 contain an intact 2 nm TCL after our usual glow discharge conditions, whereas grids from batches #1 and #2 systematically showed a broken TCL. This sensitivity was not observed on previous grid batches that were used in the same conditions. The analysis by SEM and ET clearly demonstrates the sheared state of the TCL of grids coming from batch#1 or #2 whereas no grid integrity problem, before glow discharge, could be suspected. The systematic correlation of the 3D reconstructions of glow discharge grids coming from batch#1 and their corresponding analysis by ET on grids coming from the same batch emphasizes the importance of the glow discharge parameter. The same analyses were done with nonglow discharged grids: the continuous carbon layer did not show strong defects (data not shown). As a consequence of the 2 nm TCL damage, ribosomal particles, during grid preparation, could have remained for a long time near the air-water interface which most likely explains the removal of factors (Glaeser, 2018).

The comparison between cryo-EM results from batch#3 (2 nm TCL) and other batches (2 or 3 nm TCL) suggested that the presence of an intact TCL is required for intact biological ribosomal complexes. It is worth noting that the presence of an intact TCL on batch#3 grids could not be checked as no more spare grids from batch#3 were available; this assumption was only derived from previous studies before running into the glow discharge problems and complex integrity. The other approaches used in this study could not directly correlate the TCL and the ribosomal complexes integrities.

EELS and ET estimated the thickness of the continuous carbon layer and the results obtained were in the same range as that indicated by the supplier. As demonstrated, these two techniques can quickly give some valuable checks when one needs to confirm the membrane thickness. Characterization of carbon thickness of Quantifoil grids without any extra TCL using EELS reported in previous studies (Cho et al., 2013) mentioned a thickness of 49.11 ± 8.5 nm. This is a 2-fold difference from our studies, suggesting a possible change in grid preparation. This information was not confirmed by the grid manufacturer.

Using infrared spectroscopy, the chemical evolution of the membrane could have been studied. A model (Si prism with deposited carbon) had to replace a bare grid due to signal weakness. The charge distribution and its evolution over time on a cryo-EM grid before or after glow discharge appears to be weak and poorly reproducible at the scale of the experimental sensitivity accessible in our measurements. However, it has been possible to draw a consistent picture of the chemical activation of the membrane surface and its evolution during a grid preparation time course.

Overall, we describe several approaches that can be used by cryo-EM researchers to characterize the quality of the TCL of cryo-EM grids. The variability between grid batches should always stay in mind, even though it is not an easy criterion to assess. The glow discharge parameters need also to be adapted for each batch of grids to avoid breakage of the TCL. To tackle this grid batch issue, the glow discharge parameters were lowered down to only −5 mA for 30 s for grids of batches#1 and #2 as a higher intensity above this value and time limit did still break the thin layer of carbon.

## Data Availability

The raw data supporting the conclusions of this article will be made available by the authors, without undue reservation.

## References

[B1] Aebi U. Pollard T. D. (1987). A glow discharge unit to render electron microscope grids and other surfaces H ydrop hilic. J. Electron Microsc. Tech. 7–29. 10.1002/jemt.1060070104 3506047

[B2] Berger S. D. McKenzie D. R. Martin P. J. (1988). Eels analysis of vacuum arc-deposited diamond-like films. Philos. Mag. Lett. 57, 285–290. 10.1080/09500838808214715

[B3] Cho H.-J. Hyun J-K. Kim J-G. Jeong H. S. Park H. N. You D-J. (2013). Measurement of ice thickness on vitreous ice embedded cryo-EM grids: Investigation of optimizing condition for visualizing macromolecules. J. Anal. Sci. Technol. 4, 7.

[B4] Coureux P.-D. Lazennec-Schurdevin C. Monestier A. Larquet E. Cladiere L. Klaholz B. P. (2016). Cryo-EM study of start codon selection during archaeal translation initiation. Nat. Commun. 7, 13366. 10.1038/ncomms13366 27819266 PMC5103072

[B5] Coureux P. D. Lazennec-Schurdevin C. Bourcier S. Mechulam Y. Schmitt E. (2020). Cryo-EM study of an archaeal 30S initiation complex gives insights into evolution of translation initiation. Commun. Biol. 3, 58. 10.1038/s42003-020-0780-0 32029867 PMC7005279

[B6] Egerton R. F. (2009). Electron energy-loss spectroscopy in the TEM. Rep. Prog. Phys. 72, 016502. 10.1088/0034-4885/72/1/016502

[B7] Glaeser R. M. (2018). Proteins, interfaces, and cryo-EM grids. Curr. Opin. Colloid Interface Sci. 34, 1–8. 10.1016/j.cocis.2017.12.009 29867291 PMC5983355

[B8] Godet C. David D. Sabbah H. Ababou-Girard S. Solal F. (2009). Bulk and surface plasmon excitations in amorphous carbon measured by core-level photoelectron spectroscopy. Appl. Surf. Sci. 255, 6598–6606. 10.1016/j.apsusc.2009.02.050

[B9] Gordon R. Bender R. Herman G. T. (1970). Algebraic Reconstruction Techniques (ART) for three-dimensional electron microscopy and X-ray photography. J. Theor. Biol. 29, 471–481. 10.1016/0022-5193(70)90109-8 5492997

[B10] Iakoubovskii K. Mitsuishi K. Nakayama Y. Furuya K. (2008). Mean free path of inelastic electron scattering in elemental solids and oxides using transmission electron microscopy: Atomic number dependent oscillatory behavior. Phys. Rev. B 77, 104102. 10.1103/physrevb.77.104102

[B11] Iakoubovskii K. Mitsuishi K. Nakayama Y. Furuya K. (2008). Thickness measurements with electron energy loss spectroscopy. Microsc. Res. Tech. 71, 626–631. 10.1002/jemt.20597 18454473

[B12] Iwaki M. (2002). Estimation of the atomic density of amorphous carbon using ion implantation, SIMS and RBS. Surf. Coat. Technol. 158–159, 377–381. 10.1016/s0257-8972(02)00247-5

[B13] Jahagirdar D. Jha V. Basu K. Gomez-Blanco J. Vargas J. Ortega J. (2020). Alternative conformations and motions adopted by 30S ribosomal subunits visualized by cryo-electron microscopy. Rna 26, 2017–2030. 10.1261/rna.075846.120 32989043 PMC7668263

[B14] Kazan R. Bourgeois B. Lazennec-Schurdevin C. Larquet E. Mechulam Y. Coureux P-D. (2022). Role of aIF5B in archaeal translation initiation. Nucleic Acids Res. 50 (11), 6532–6548. 10.1093/nar/gkac490 35694843 PMC9226500

[B15] Lu X. Naidis G. Laroussi M. Reuter S. Graves D. Ostrikov K. (2016). Reactive species in non-equilibrium atmospheric-pressure plasmas: Generation, transport, and biological effects. Phys. Rep. 630, 1–84. 10.1016/j.physrep.2016.03.003

[B16] Malis T. Cheng S. C. Egerton R. F. (1988). EELS log‐ratio technique for specimen‐thickness measurement in the TEM. J. Electron Microsc. Tech. 8, 193–200. 10.1002/jemt.1060080206 3246607

[B17] Mastronarde D. N. (2005). Automated electron microscope tomography using robust prediction of specimen movements. J. Struct. Biol. 152, 36–51. 10.1016/j.jsb.2005.07.007 16182563

[B18] Mastronarde D. N. (1997). Dual-axis tomography: An approach with alignment methods that preserve resolution. J. Struct. Biol. 120, 343–352. 10.1006/jsbi.1997.3919 9441937

[B19] Mechulam Y. Guillon L. Yatime L. Blanquet S. Schmitt E. (2007). Protection-based assays to measure aminoacyl-tRNA binding to translation initiation factors. Methods Enzymol. 430, 265–281. 10.1016/S0076-6879(07)30011-6 17913642

[B20] MessaoudiI C. Boudier T. Sorzano C. O. S. Marco S. TomoJ. (2007). Tomography software for three-dimensional reconstruction in transmission electron microscopy. BMC Bioinforma. 8, 1–9. 10.1186/1471-2105-8-288 PMC1976622 17683598

[B21] Noble A. J. Dandey V. P. Wei H. Brasch J. Chase J. Acharya P. (2018). Routine single particle CryoEM sample and grid characterization by tomography. Elife 7, e34257. 10.7554/eLife.34257 29809143 PMC5999397

[B22] Passmore L. A. Russo C. J. (2016). Specimen preparation for high-resolution cryo-EM. Methods Enzym. 579, 51–86. 10.1016/bs.mie.2016.04.011 PMC5140023 27572723

[B23] Querido J. B. Sokabe M. Kraatz S. Gordiyenko Y. Skehel J. M. Fraser C. S. (2020). Structure of a human 48S translational initiation complex. Science 369, 1220–1227. 10.1126/science.aba4904 32883864 PMC7116333

[B24] Rao A. V. Chazalviel J.-N. Ozanam F. (1986). *In situ* characterization of the n-Si/acetonitrile interface by electromodulated infrared internal-reflection spectroscopy. J. Appl. Phys. 60, 696–706. 10.1063/1.337417

[B25] Russo C. J. Passmore L. A. (2016). Progress towards an optimal specimen support for electron cryomicroscopy. Curr. Opin. Struct. Biol. 37, 81–89. 10.1016/j.sbi.2015.12.007 26774849 PMC4863039

[B26] Scheres S. H. W. R. E. L. I. O. N. (2012). Relion: Implementation of a bayesian approach to cryo-EM structure determination. J. Struct. Biol. 180, 519–530. 10.1016/j.jsb.2012.09.006 23000701 PMC3690530

[B27] Zhang K. Gctf (2016). Gctf: Real-time CTF determination and correction. J. Struct. Biol. 193, 1–12. 10.1016/j.jsb.2015.11.003 26592709 PMC4711343

[B28] Zheng S. Q. Palovcak E. Armache J. P. Verba K. A. Cheng Y. Agard D. A. (2017). MotionCor2: Anisotropic correction of beam-induced motion for improved cryo-electron microscopy. Nat. Methods 14, 331–332. 10.1038/nmeth.4193 28250466 PMC5494038

