# Supplementary material for: Grid batch-dependent tuning of glow discharge parameters
Source: Front Mol Biosci. 2022 Aug 18;9:910218. doi: 10.3389/fmolb.2022.910218 (PMC9436422; doi:10.3389/fmolb.2022.910218)
Supplement: Supplementary file 1 [file DataSheet1.PDF]

SUPPLEMENTARY FIGURES

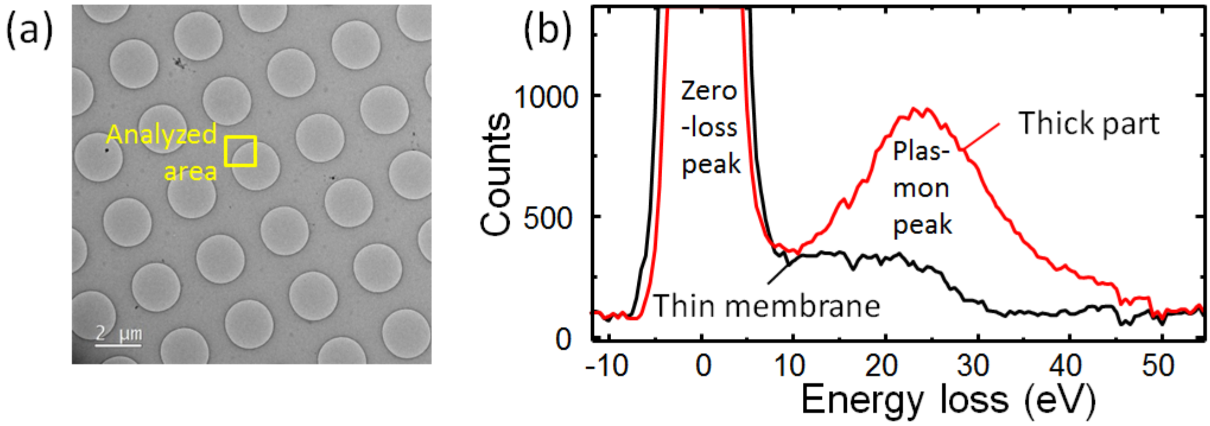

Supplementary Figure S1: a) TEM general view of a carbon membrane: thick part in dark grey and disk-shape thin parts in light grey; some dust – not present on all membranes – is visible. The yellow square indicates the type of area in which the analyses of Figure 3 are recorded. b) EEL spectra recorded in a thin part (black) and in a neighboring thick part (red); note the standard shape of the thick-part spectrum, with the a-C plasmon peak at 24 eV, in contrast with the specific shape of the thin-part spectrum, where the plasmon losses are shifted towards low energies.

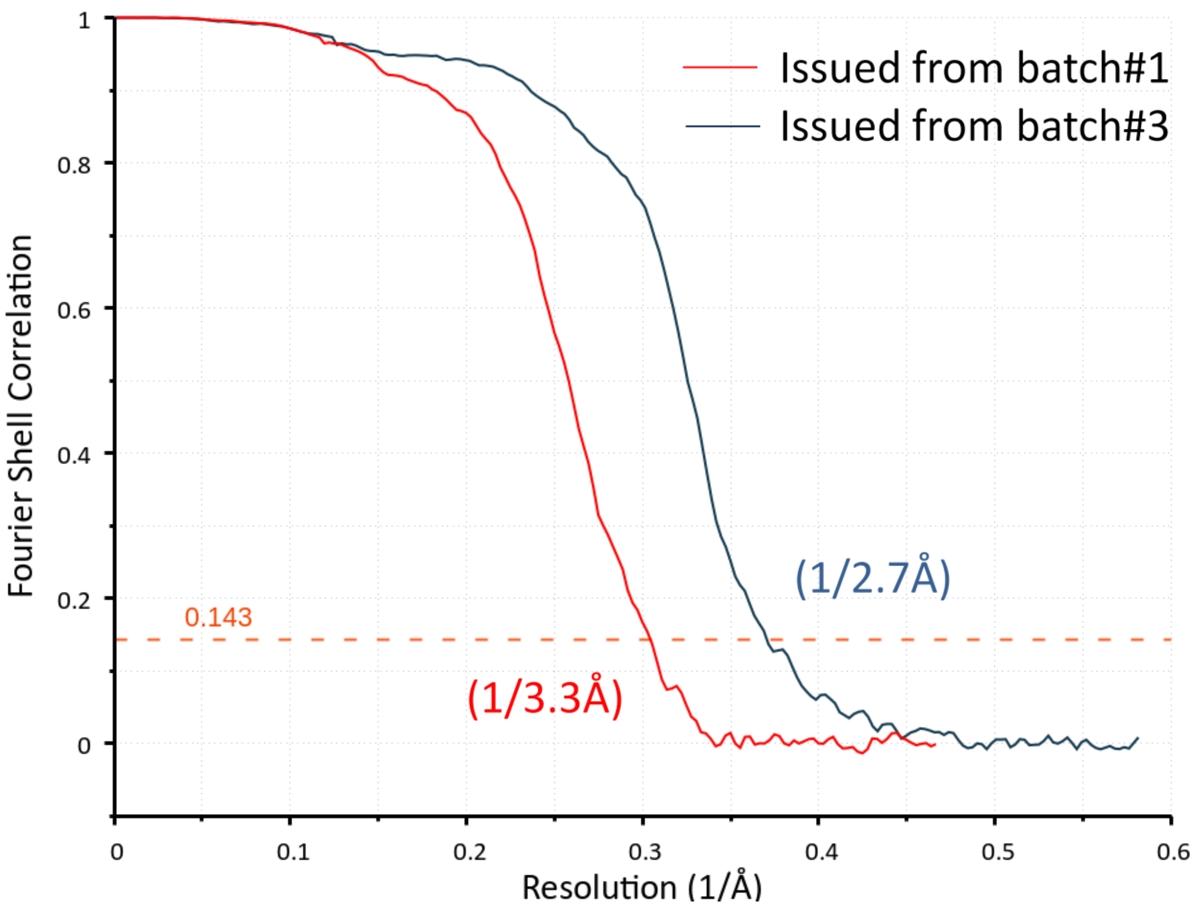

Supplementary Figure S2: FSC curves of potiental maps obtained with data collected on grids from batch#1 (in red) and batch#3 (in blue). The limit of resolution of the 3D reconstructions obtained for batch#1 and batch#3 and measured by the gold-standard FSC in Relion 3.1 was estimated at 3.3 Å and 2.7 Å, respectively.

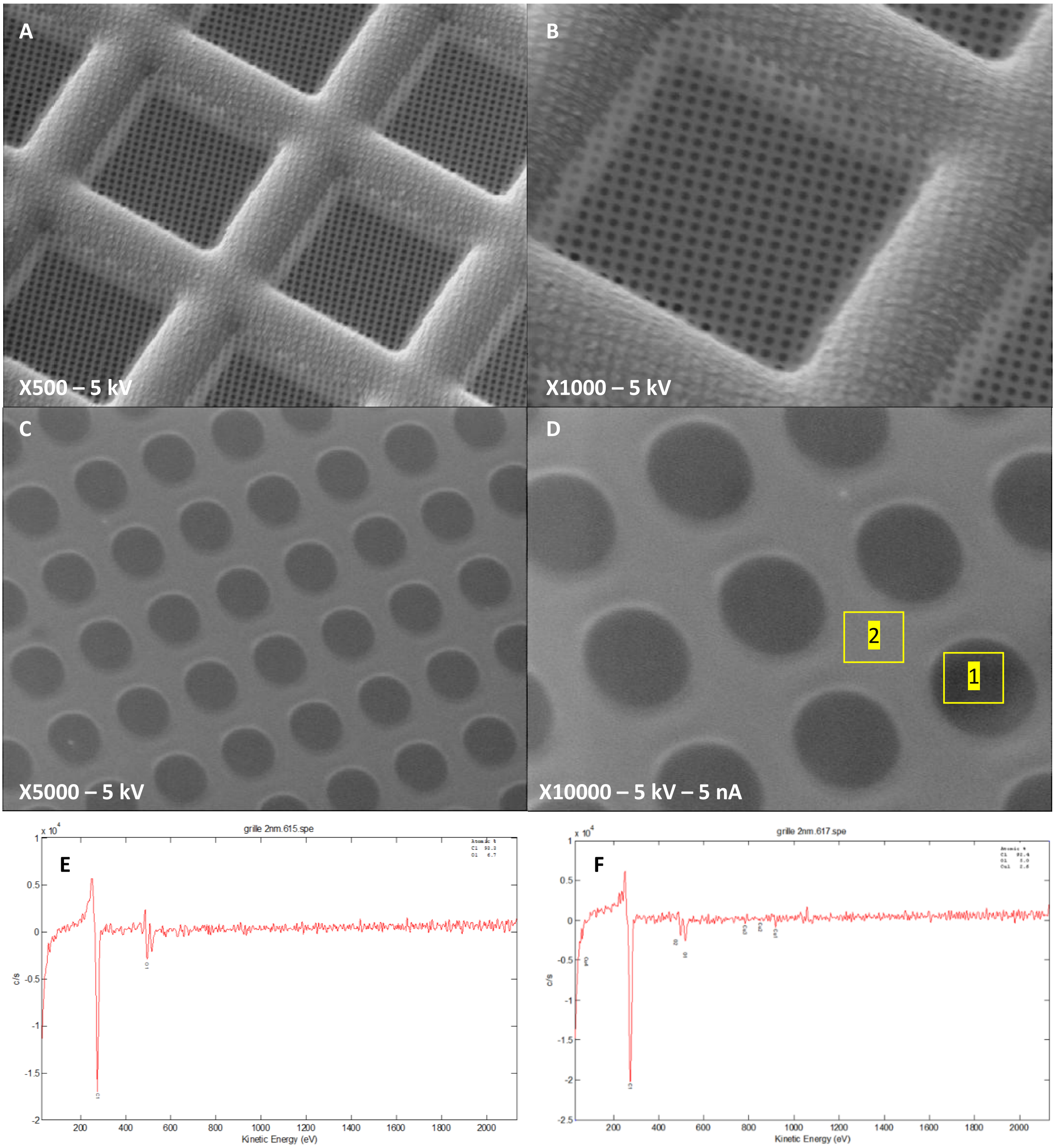

Supplementary Figure S3: Auger Electron Spectroscopy on 2 nm continuous carbon layer grid (batch #1). (A) Image of the grid at 500x magnification. (B) Image of the grid at 1000x magnification. (C) Image of the grid at 5000x magnification. (D) Image of the grid at 10000x magnification with a probe current of 5 nA. The position 1 (inside the hole) and 2 (outside the hole) that were probed are drawn in yellow. (E) Auger analysis inside hole (position 1) : C: 93,3%; O:6,7% (F) Auger analysis outside hole (position 2) : C:92,4%,O:5%,Cu 2,6%. Similar results were obtained for batch #2.

Remark: the Cu Auger signature is probably due to backscattered electrons effect on the grid

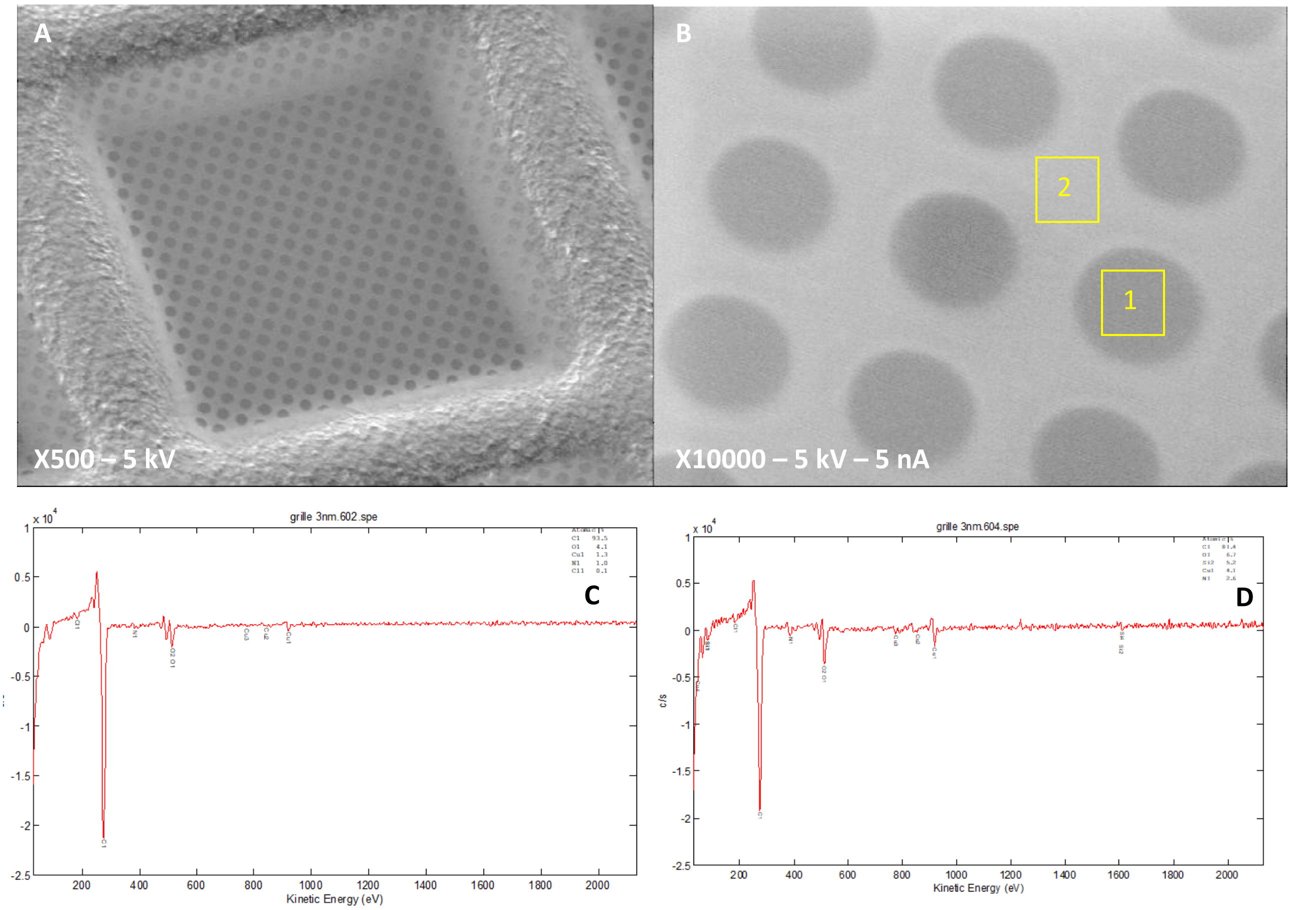

Supplementary Figure S4: Auger Electron Spectroscopy on 3 nm continuous carbon layer grid (batch #4). (A) Image of the grid at 500x magnification. (B) Image of the grid at 5000x magnification with a probe current of 5 nA. The position 1 (inside the hole) and 2 (outside the hole) that were probed are drawn in yellow. (C) Auger analysis inside hole (position 1) : C: 93,5%; O:4,1%;Cu,N,Cl (D) Auger analysis outside hole (position 2) : C: 81,4%,O:6,7%,Si:5,2%Cu:4,1% N:2,6% traces of Cl

Remark: the Cu and Si Auger signature is probably due to backscattered electrons on the grid outside the hole and/or local pollution.

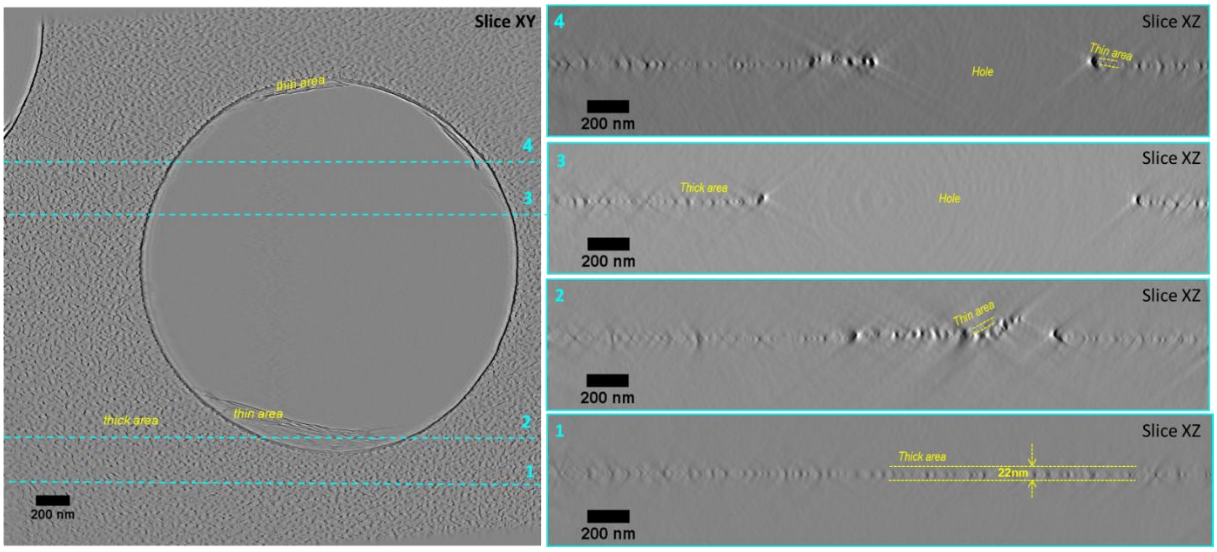

Supplementary Figure S5: Electron tomography analyses of a second area of the Quantifoil membrane containing a thick and thin area. Transversal (left) and longitudinal (right) slices acquired at different depths and orientations through the reconstructed volume, illustrating the roughness of the Quantifoil membrane.

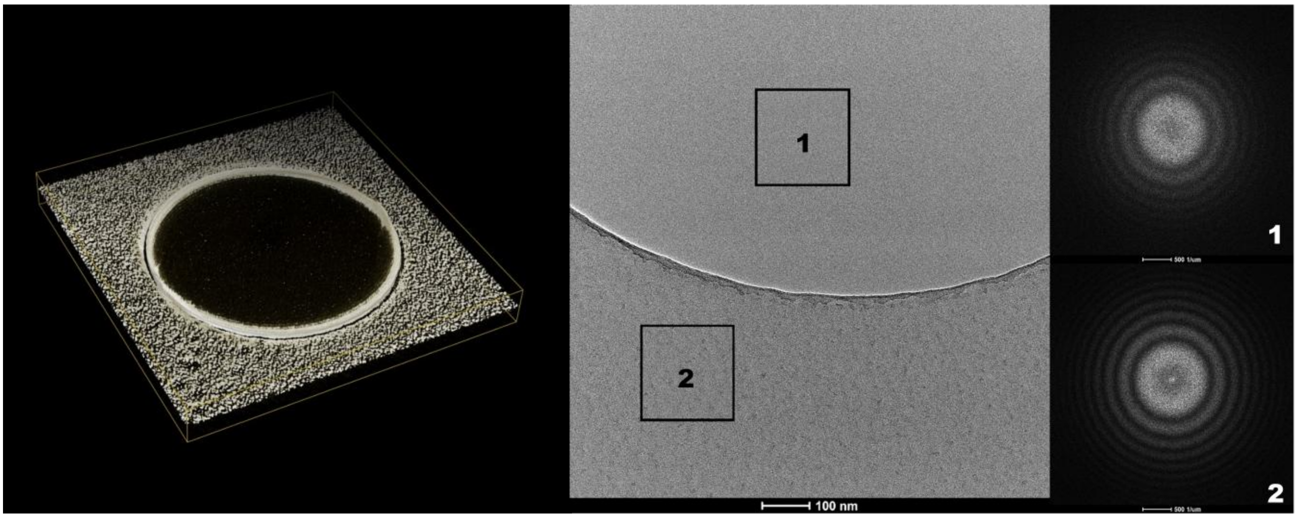

Supplementary Figure S6: Electron tomogram obtained on an intact 2 nm continuous carbon layer grid (batch #1). The power spectrum of thin (position 1) or thick (position 2) areas was computed to assess the presence or absence of a carbon layer.

Nominal thickness	Record number	t/λ	Thickness t taking λ = 130 nm (nm)
2 nm	1	0.0299	3.9
	2, thinner part	0.0392	5.1
	2, thicker part	0.0522	6.8
	3	0.0288	3.7
	4	0.0425	5.5
	5	0.0287	3.7
Average			4.8
Standard deviation			1.2
3 nm	1	0.0370	4.8
	2	0.0420	5.5
	3	0.0448	5.8
	4	0.0388	5.0
	5	0.0417	5.4
Average			5.3
Standard deviation			0.4

Supplementary Table 1: Thickness measured by TEM-EELS. For the 2 nm case, one may see two values of the measurement labelled "2": they have been obtained in respectively the thinner and thicker parts of the profile shown in black in Figure 3b. These values are quite high, not really typical of this membrane, but characteristic of the large departures to the nominal thickness that exist in it. Such departures explain the high standard deviation, three-times as large in the 2 nm case compared to the 3 nm one.
